# 1798. Vaccine Ambassadors: An Educational Model to Spread Awareness About Vaccines in Detroit

**DOI:** 10.1093/ofid/ofad500.1627

**Published:** 2023-11-27

**Authors:** Jennifer Schmidt, Sofia Howson, Catherine Maples, Jack McConnell, Soham Desai, Teena Chopra, Matthew Seeger

**Affiliations:** Wayne State School of Medicine, Belleville, Michigan; Wayne State School of Medicine, Belleville, Michigan; Wayne State School of Medicine, Belleville, Michigan; Wayne State School of Medicine, Belleville, Michigan; Wayne State School of Medicine, Belleville, Michigan; Detroit Medical Center, Wayne State University, Detroit, MI; Wayne State University, Detroit, Michigan

## Abstract

**Background:**

Since the COVID-19 pandemic, national vaccination rates show a dangerous decline with Detroit being amongst the lowest with only 41.3% of adolescents completing the vaccine series. The Vaccine Ambassador program was created to mitigate the decline in vaccination rates through the education and empowerment of youth via train-the-trainer model of education.

**Methods:**

The program was implemented with eleven high school students who received education on the history and mechanism of vaccines, herd immunity, and how to effectively communicate. The program was created in collaboration with physicians in Infectious Diseases and a professor of communication at Wayne State University.

Ambassadors aimed to spread this knowledge to youth in Detroit communities through outreach events. The impact of the program was measured through a two-pronged approach—through a pre- and post-survey given to the Vaccine Ambassadors and through a pre- and post-test given to the youth at the events. The survey for the Ambassadors included thirty-one items that measured their knowledge about vaccines and immunity and were evaluated through a 5-point Likert scale from 1 (strongly disagree) to 5 (strongly agree). The test for the youth at the outreach events included seven items that tested knowledge about vaccines.

**Results:**

The following prompt is an example from the survey given to the ambassadors: Inactive vaccines create a better immune response than live vaccines. Compared to the pre-survey, six participants (54.5%) indicated a less agreeable response. With regards to the youth outreach events, twenty-five youth participated in the pre- and post-test, and nineteen youth (76.0%) improved their score after the presentation. Post-test scores improved across all seven questions (Figure 1).

Comparison of Pre and Post Test Responses During Outreach Events

**Figure:**
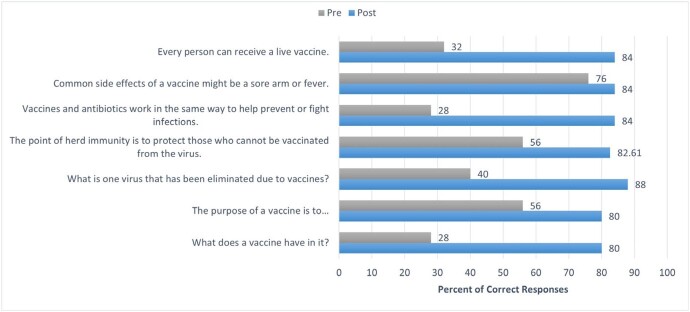

Representation of the percent correct questions before and after the Vaccine Ambassador presentation

**Conclusion:**

Based on both pre-test responses, it is apparent that Detroit’s youth lack knowledge regarding vaccines. The Vaccine Ambassador program increased the ambassador’s depth of knowledge and reaffirmed their positive attitudes about vaccines. Not only are youth able to learn, but they are able to apply their knowledge and teach others. By teaching their peers, youth can make informed decisions when older and enact behavioral changes that promote healthier living.

**Disclosures:**

**All Authors**: No reported disclosures

